# Function by Structure: Spotlights on Xist Long Non-coding RNA

**DOI:** 10.3389/fmolb.2017.00090

**Published:** 2017-12-19

**Authors:** Greta Pintacuda, Alexander N. Young, Andrea Cerase

**Affiliations:** ^1^Department of Biochemistry, University of Oxford, Oxford, United Kingdom; ^2^European Molecular Biology Laboratory, Monterotondo, Italy

**Keywords:** xist RNA, RNA-structure, epigenetics, X chromosome inactivation, RNA-protein interaction, 3D-organization

## Abstract

Recent experimental evidence indicates that lncRNAs can act as regulatory molecules in the context of development and disease. Xist, the master regulator of X chromosome inactivation, is a classic example of how lncRNAs can exert multi-layered and fine-tuned regulatory functions, by acting as a molecular scaffold for recruitment of distinct protein factors. In this review, we discuss the methodologies employed to define Xist RNA structures and the tight interplay between structural clues and functionality of lncRNAs. This model of modular function dictated by structure, can be also generalized to other lncRNAs, beyond the field of X chromosome inactivation, to explain common features of similarly folded RNAs.

## Introduction

For many years the scientific community has been divided on whether lncRNAs represent non-functional transcriptional noise or important regulatory elements (Blake et al., 2003; Ponjavic et al., 2007). Recent work in cell lines and mouse models supports the hypothesis that few lncRNAs are important mediators of cellular functions regulating different levels of gene expression (Sauvageau et al., 2013; Ramos et al., 2015; Engreitz et al., 2016b; Liu B. et al., 2017). LncRNAs have been shown to work on four regulatory levels: (1) as macromolecular scaffolding for protein recruitment (Cerase et al., 2015; McHugh et al., 2015; Minajigi et al., 2015; Pinter, 2016); (2) as molecular sponges for sequestering regulatory ncRNAs or proteins (Cesana et al., 2011; Wu et al., 2017); (3) as a genomic 3D organizer (Hacisuleyman et al., 2014; Cerase et al., 2015); (4) as *cis/trans*-regulatory elements regulating transcription and RNA-splicing (Engreitz et al., 2016a; Wu et al., 2017).

One of the best-studied examples of lncRNAs is Xist (*X inactive specific transcript*). Xist is the master regulator of X chromosome inactivation (XCI) and it is both necessary and sufficient for establishing this process, which results in the stable and efficient silencing of one X chromosome of somatic cells of female mammals early in development (Cerase et al., 2015). Xist is known to act as scaffolding for protein recruitment, as well as an organizer of the inactive X chromosome (Xi) in 3D-space (Splinter et al., 2011; Cerase et al., 2015; Pintacuda and Cerase, 2015; Giorgetti et al., 2016; Pinter, 2016). How Xist mediates these cellular functions through its associated proteins is still debated. Recent genetic and biochemical work revealed a complex Xist interactome consisting of hundreds of potential interactions (Chu et al., 2015; McHugh et al., 2015; Minajigi et al., 2015; Moindrot et al., 2015; Monfort et al., 2015). Subsequent work has predicted that over 30 RNA-binding proteins directly interact with Xist (Cirillo et al., 2016). Among these, SAF-A was shown to mediate Xist anchoring to the nuclear matrix (Hasegawa et al., 2010); RMB15/RBM15b were implicated in the m6A pathway responsible for Xist post-transcriptional modification (Patil et al., 2016); hnRNPK is required for Polycomb recruitment and subsequent deposition of repressive histone modifications (such as H3K27me3 and H2A119ub) (Chu et al., 2015; Almeida et al., 2017; Pintacuda et al., 2017); Spen (also known as SHARP/MINT) is necessary for the establishment of primary gene silencing; while Lbr is required to anchor the inactive chromosome (Xi) to the nuclear periphery, establish and stabilize gene silencing during the maintenance phase of X inactivation (Chen et al., 2016) (explained in more details below). The precise binding sites and mechanisms of action of the majority of these proteins are unknown (Chu et al., 2015; Chen et al., 2016; Van Nostrand et al., 2016). 3D structural studies of these protein-RNA interactions have yet to be explored.

**Figure 1:**
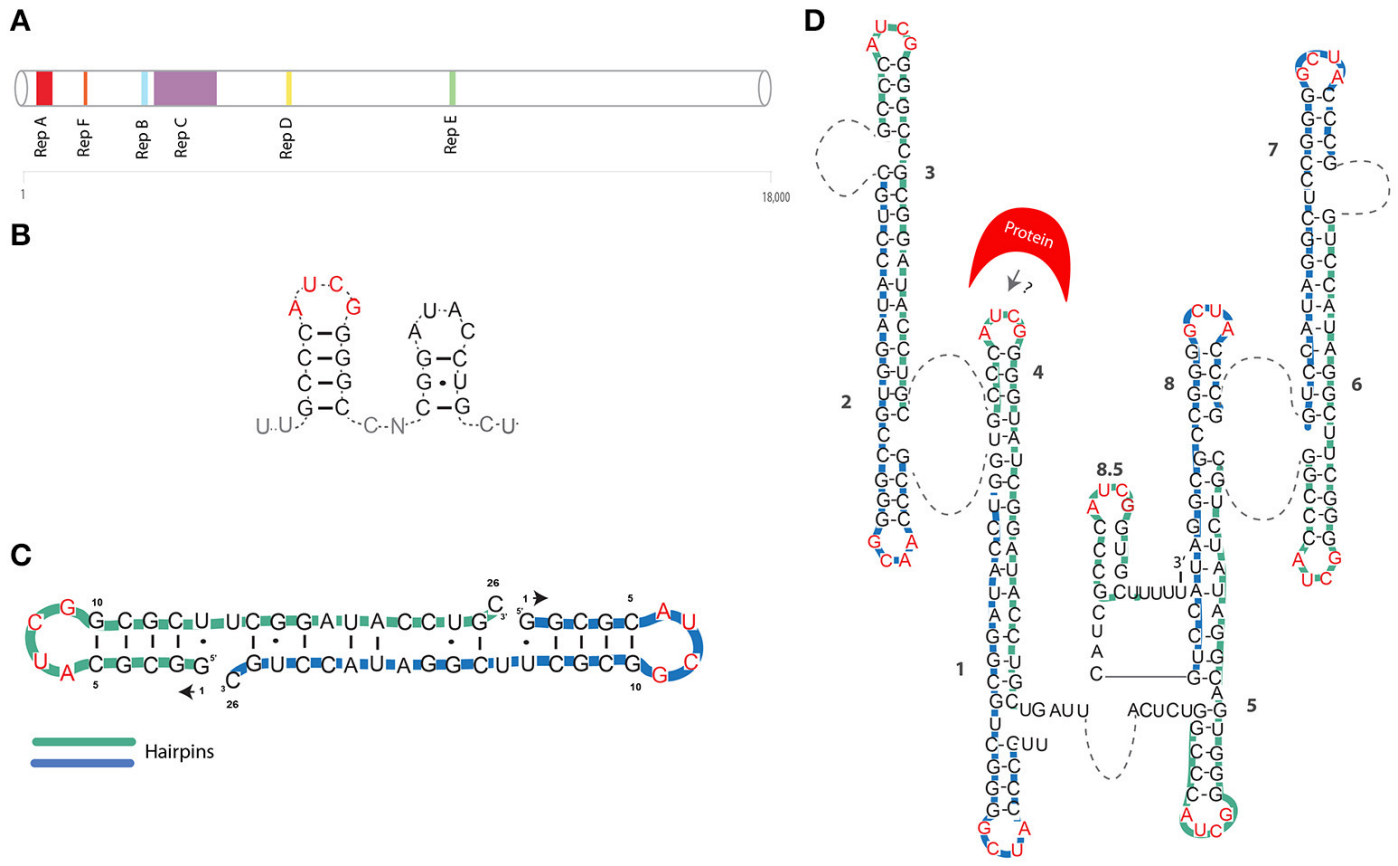
**(A)** Position of tandem repeats is shown for the Xist mouse transcript (Brockdorff et al., 1992). **(B)** The first proposed model of Xist A-repeat structure (Wutz et al., 2002). **(C)** First NMR-based model of a single A-repeat (Duszczyk et al., 2008). **(D)** NMR/mutational model of the XIST A-repeat (Duszczyk et al., 2011). Green and blue lines represent individual repeats. AUCG tetraloops are shown in red. Dashed lines represent unstructured U-rich linkers.

It is known that Xist-protein interactions are mostly mediated by the structured regions of Xist RNA (Chu et al., 2015; Fang et al., 2015; Chen et al., 2016; Lu et al., 2016; Smola et al., 2016), or Xist motifs (Smola et al., 2016). In mouse, Xist has six conserved regions of tandem repeats, named A to F, that are essential for its function (Brockdorff, 2002; Wutz et al., 2002) (Figure 1A). Xist tandem repeats are conserved in mammalian vertebrates. All mouse repeats have conserved human XIST counterparts, however considerable variation in the copy number of the repeats is observed, with the exception of the A-repeat region, which is conserved both in terms of copy number and consensus sequence, and is likely to mediate most of the interactions resulting in early gene silencing (Wutz et al., 2002; Patil et al., 2016). Conservation outside the repeats is relatively poor (Nesterova et al., 2001).

In this review we systematically analyse the results of the experiments that have been carried out to characterize Xist RNA structure, in order to put structural information in the context of a genetic and biochemical analysis of Xist function.

## Xist topology and RNA structures

### Xist A-repeats

The foundations of Xist RNA structural analyses were laid by a seminal study, where Wutz and colleagues created a series of deletions spanning most of the Xist sequence, using inducible Xist cDNA systems (Wutz et al., 2002). This study, showed that a 5' conserved region, named A-repeat (or RepA), was indispensable for gene-silencing (Figure 1A). The Xist A-repeat is highly conserved in mammals, in mouse it consist of 7.5 copies (8.5 in humans) of 26-mers separated by U-rich linkers (Brockdorff, 2002). Using a reporter system, the authors also showed that the number of repeats is directly linked to the efficiency of silencing. By mutagenesis analysis, Wutz et al. further inferred that the structure, rather than the sequence, of the stem and loop was crucial for silencing (Wutz et al., 2002). They suggested that this 26-mer was likely to assume a double stem-loop structure (Figure 1B).

In 2008 NMR studies by Duszczyk et al., showed that the 26-mer of the A-repeat are unlikely to fold into two separate stem-loops (Duszczyk et al., 2008) (Table 1). A single repeat is likely to form only the major stem-loop structure suggested by Wutz and colleagues. Notably, this RNA hairpin with an AUCG loop sequence was found to be thermodynamically highly stable, comparable to other so-called “stable” tetraloops. This suggests that this hairpin could be a basic folding unit of the A-repeat region. In contrast, the second stem-loop is likely to be involved in inter-repeat dimerization (Figure 1C). In 2011, the Sattler group used a combination of mutagenesis and NMR analysis to propose a model in which the 8.5 copies of the human XIST A-repeat form a series of inter and intra-repeat bindings, resulting in several exposed AUCG tetraloops connected by U-rich linkers (Duszczyk et al., 2011) (Figure 1D and Table 1). The authors suggested that these structures may function as a multimerization platform for protein binding. This model envisioned for the first time inter-loop dimerization of non-consecutive individual A-repeats. In detail, it was suggested that repeats 1–4, 2–3, 6–7, and 5–8 form inter repeat dimers, generating a unique 2D structure of the A-repeat region (Figure 1D).

**Table 1:** Methods to study RNA structure and RNA-protein interactions.

**NMR**
Nuclear magnetic resonance spectroscopy can be a very powerful tool for studying RNA structures in fine detail with a high confidence. It is advantageous to X-Ray crystallography techniques as RNA molecules can be studied in a more natural state while dissolved in solution, however it relies on large preparations of highly pure and uniform RNA and is generally restricted to solving small discrete structures.
**PARS**
This technique brings together classic RNA footprinting techniques with next-generation sequencing. It involves treating RNA independently RNase V1 and S1 nucleases which cut double and single stranded RNA respectively. Cleaved fragments are adaptor ligated and sequenced allowing a map to be generated of single and double stranded RNA down to single nucleotide resolution.
**DMS**
DMS treatment modifies RNA by adding a methyl group to any unpaired or loosely structured A and C bases in a sample. Once methylated the bases can no longer form base pairs and will cause cDNA transcripts to terminate early. When compared to a non-DMS treated sample the sites of early termination, and thus the presence of unpaired bases can be deduced. The addition of next generation sequencing (DMS-seq/Structure-seq) greatly increases the power of the technique and allows the rates of base modification to be mapped in a quantitative manner. Targeted Structure-seq improves the specificity and power of the technique by using primers targeted for the length of a specific RNA of interest instead of sequencing the whole transcriptome.
**SHAPE**
SHAPE methods use chemical reagents which selectively modify flexible or unpaired bases by forming adducts on the 2'-hydroxyl of the RNA backbone. As with other modifications these adducts will result in the early termination reverse transcription. As the reagents only modify the RNA backbone, they have the advantage of being independent of base identity and provide a reliable measurement of individual nucleotide flexability. SHAPE-Map uses specialized conditions for reverse transcription which result in the misreading of SHAPE-modified nucleotides and the introduction of non-complementary base mutations instead of early termination. These mutations are easily identified after sequencing and their relative frequencies can be mapped to the reference sequence.
**PARIS**
PARIS works by fixing the base pairs of dsRNA of cells *in vivo* using the specific and reversible nucleic acid cross-linker called AMT. After cross-linking, samples undergo proteinase treatment and partial RNA degradation. Subsequently they are gel purified from a 2D gel, leaving only fixed dsRNAs. dsRNA then undergoes proximity ligation and undergo next generation sequencing. The resulting reads represent all the native dsRNA in the organism and can be mapped to infer their structure.
**RIP**
RNA immunoprecipitation takes advantage of antibodies to pull down RNA bound to a given protein. The technique cannot differentiate between direct and indirectly bound RNA and may also generate false positives from interactions that occur after cell lysis.
**CLIP**
Improves the specificity of RIP by UV crosslinking of RNA/protein complexes before extraction. This allows the removal of weakly bound RNA through stringent washing. The remaining RNA can then undergo reverse transcription and PCR amplification (or next generation sequencing). The main drawback of this method is the loss of a significant proportion of transcripts which are stalled at the cross-linking site resulting in truncated cDNAs. UV crosslinking can also introduce some bias as its ability to bind RNA to protein varies depending on the base/aa mediating the interaction.
**iCLIP**
Individual-nucleotide-resolution CLIP (iCLIP) was developed to enable recovery of truncated cDNAs lost in conventional CLIP. This is achieved by the circularization of cDNA after reverse transcription, attaching a new barcoded adaptor to the truncated end allowing it to be amplified after linearization. Barcode filtering allows truncated cDNAs to be identified along with the crosslinked nucleotide. Subsequent mapping of these small fragments to the reference sequence can be difficult.
**eCLIP**
Enhanced CLIP improves library preparation and circular ligation steps of iCLIP allowing greater power in filtering and mapping truncated sequences.

*For more details on these techniques, we refer the reader to the following reviews (Shen et al., 1995; Latham et al., 2005; Low and Weeks, 2010; Leone and Santoro, 2016; Somarowthu, 2016; Barra and Leucci, 2017)*.

**Figure 2:**
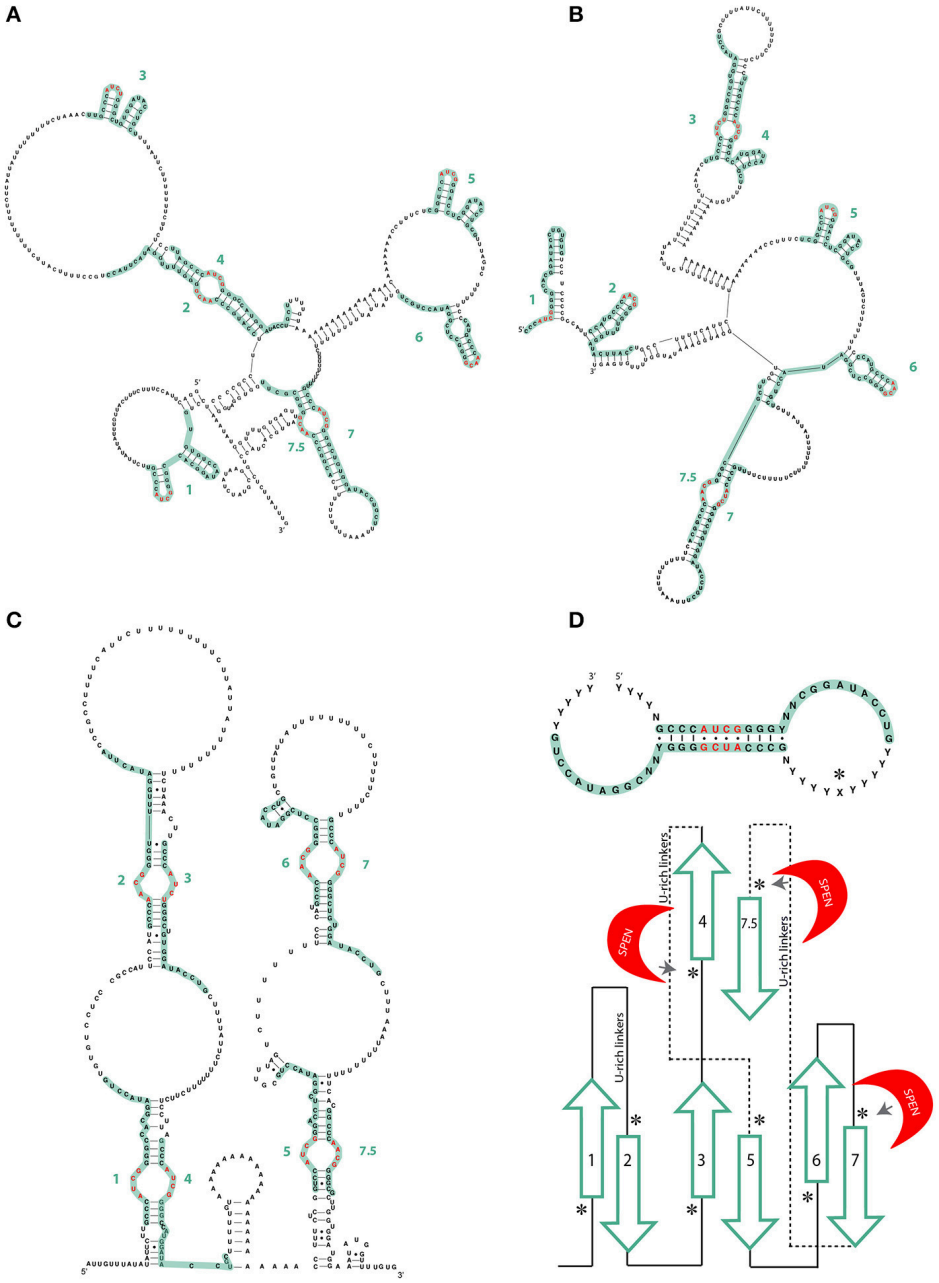
**(A)** Different models of the mouse A-repeat of Xist by Fang et al. (2015) and **(B)** Liu N. et al. (2017). Green lines represent individual repeats. AUCG tetraloops are shown in red. **(C)** Mouse Xist Rep A model by Maenner et al. (2010). There are no significant differences from human XIST. Green lines represent individual repeats. AUCG tetraloops are shown in red. **(D)** Lu et al. model of the consensus inter-repeat structural unit and the repeat pairing of the mouse Xist A-repeat region. Paired repeats are shown in green, U-rich linkers are shown as lines. SPEN crosslinking sites as determined by iCLIP are indicated by an asterisk (Lu et al., 2016).

At a similar time, Maenner and colleagues, using a combination of chemical and enzymatic probing of the full-length human and mouse A-repeat elements, proposed three theoretical models that could account for the physiological folding of the A-repeats *in vivo* (Maenner et al., 2010). Subsequent fitting of FRET experiments lead to ultimate selection of only one model. The selected model suggests that the A-repeat consists of two major double stem-loops, separated by a smaller stem-loop domain, with no major differences between mouse and human (Figure 2C, for clarity, we will only discuss the selected model). Similar to the model proposed by the Sattler group (Duszczyk et al., 2011), the authors suggest that repeats 1–4, 2–3, 6–7, and 5–8 dimerize. A noticeable difference between the proposed structures, is that Duszczyk et al. suggest the AUCG tetraloop is exposed as an apical loop, while Maenner and colleagues propose that it forms an internal bulge (Figures 1D, 2C respectively).

In 2015 and 2017, two additional papers studying the Xist A-repeat structure were published, both making use of experimental techniques to directly infer Xist structure rather than modeling (Fang et al., 2015; Liu F. et al., 2017). Fang et al. used Targeted Structure-seq, a combination of *in vivo* DMS chemical probing and next-generation sequencing. Liu et al. performed SHAPE and *in vitro* DMS probing on samples prepared using a non-denaturing purification protocol that ensured high purity and homogeneity (Chillón et al., 2015) (Table 1). The A-repeat structures obtained are similar, differing primarily in the pairing of A-repeats. A-repeats 2–4, 7–8, form inter repeat dimers in the structure from Fang et al.; 3–4 and 7–8 form dimers in the structure from Liu and colleagues. Nevertheless, they have strikingly similar stem-loop structures, both emerging from larger RNA bulges of repeats 3, 5, and 6 (Figures 2A,B). These differences may be explained by differences in protein binding (see Function by Structure paragraph).

Xist A-repeat structure was also studied through a novel technique named PARIS (Lu et al., 2016). As a part of this method, RNA base pairs are cross-linked *in vivo*, the dsRNA is recovered and then subjected to next generation sequencing. The resulting reads of these ligated RNA “duplexes” represent all the native dsRNA in the organism and can be mapped to infer their structure(s) (Table 1). Data from Lu et al. also supported the model of an isolated A-repeat domain, with the inter-repeat conformation being more likely to form and being more stable as opposed to the intra-repeat structures proposed by Wutz et al. (2002). As with the model proposed by Maenner and colleagues and in contrast to that proposed by Duszczyk et al. (2011) the basic structural unit of the inter-repeat dimer has the AUCG tetraloop as internal to the dimerization region (see Figures 1C, 2D). This basic structural unit of the inter-repeat binding is also evident in the structures presented by Fang et al. and Liu et al. (Figures 2A,B). Interestingly, Lu et al. did not find a single solution to the 2D structure of the A Repeat region and suggested that Xist lncRNA is likely to have a dynamic structure presenting many different conformations, while still maintaining an overall functional structure. These observed differences between *in vitro* and *in vivo* studies could also potentially be explained by the presence of proteins interacting with the RNA structure *in vivo*.

All aforementioned studies support the notion that the structure of lncRNAs, is conserved during evolution and defines biological function. However, a recent study, based on statistical and phylogenetic analysis, suggests that the function of lncRNA is mostly sequence dependent, as there is no clear indication for structural conservation. One of the lncRNAs analyzed in this study was Xist (Rivas et al., 2017); the authors propose that Xist may primarily function through its primary structure. However, this view contrasts with the biological evidence that Xist interacting partners localize predominantly to those repeats where a secondary structure was predicted (see Function by Structure paragraph), or direct perturbation of structure was shown to lead to loss of Xist function (Wutz et al., 2002). Semi-stochastic inter-repeat pairing could also explain this apparent lack of conservation (Lu et al., 2017).

### Full-length Xist structures

Recently two groups obtained *in vivo* structures of Xist RNA molecules (Fang et al., 2015; Smola et al., 2016). Fang and colleagues, combining DMS-sensitivity assays with next-generation sequencing, obtained the very-first *in vivo* full length (FL) secondary structure of Xist RNA (Table 1). Noticeably, the DMS-profile underlying structured regions is in good agreement with the predicted thermodynamically stable structures of Xist RNA. Thermodinamically stable regions correlate well with RNA structured regions.

Using a variant of SHAPE technology, called SHAPE-MaP, Smola and colleagues obtained an *in vivo* Xist structure (Smola et al., 2016) (Table 1). In this context, the A-repeat seems to be highly structured while the E-repeat is loosely structured. In the case of the E-repeat, an RNA motif seems to sustain most interactions rather than any clear secondary structure. Interestingly the authors also reported a novel Xist 3' structured region, in agreement with previous predictions and observations, including those from Fang et al. (2015). Their *in vivo* SHAPE profile, is in line with the predicted thermodynamically stable structures of Xist RNA. This work however, failed to map the B- and C-repeat of Xist (see also Function by Structure paragraph).

Finally, a new high-throughput algorithm was implemented to profile RNA structure. This algorithm called CROSS has been trained on existing SHAPE, PARS and NMR datasets (Delli Ponti et al., 2017). Xist structure generated by CROSS is in very good agreement with the experimental data of Smola et al. (2016). Noticeably, also this study predicts the presence of structured regions at Xist 3'-end that may be important for its localization (Yamada et al., 2015).

## Function by structure

Proteins exert their regulatory function by exploiting the thermodynamic properties of their environment. In this perspective, their tertiary structures provide the interaction interface with the environment, and therefore dictate affinity for ligands (protein, nucleic acids, small molecules) or enzymatic activity. Similarly, RNA can exert its biological function by adopting discrete 3D-folding. However, differently from proteins, the tertiary folding of RNA is thought to be based on the initial formation of stable secondary structures, building hierarchical blocks (Bailor et al., 2011). Although a systematic catalog of regulatory RNA folds has not been published to date, several well-defined secondary structures have been described to recur in many regulatory RNAs (Bhartiya et al., 2013) (see also Rfam websites and RNA Central; http://rfam.xfam.org/; http://rnacentral.org/). Among these are double stem loop motifs, broadly associated with chromatin remodeling; cloverleaf-like architectures, originally described for tRNAs, and generally found in lncRNAs involved in 3′ end processing, such as MALAT1 and NEAT1; and pseudoknots, mostly found in catalytic RNAs, such as the RNA components of telomerases. Interestingly, it appears that RNA secondary structures within the same lncRNA, tend to form modular platforms (Somarowthu et al., 2015). In this perspective, the modular structure of the subsequent repetitive regions of Xist RNA, represents a good model to study the functional integration of an array of independent domains.

### Xist tandem repeats and binding proteins

#### A-repeat

Work by Lu et al., in which the PARIS method is applied to Xist RNA, defines the first mechanistic model of interaction of Xist with the silencing factor SPEN (also known as SHARP/MINT) (Lu et al., 2016). The model proposed by the authors suggests that SPEN scans Xist in a sequence-independent manner but nucleates only at the A-repeat region. Specifically, the long-range inter-repeat helices formed by the A-repeat create multiple copies of a duplex structure. These helices are flanked by U-rich sequence motifs bound by SPEN RRM domains—although a role of the structured regions of the A-repeat stabilizing such interaction cannot be formally excluded. Previously published crystallographic *in vitro* data supports this conclusion (Arieti et al., 2014).

**Figure 3:**
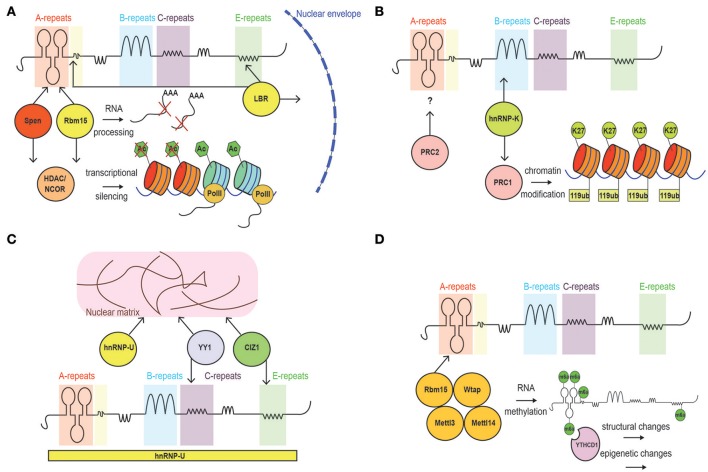
Schematic of Xist interacting proteins. **(A)** Factors involved in the establishment of Xist silencing. These include Ncor/histone deacetylase-interacting proteins, and LBR, which tethers the inactive chromosome to the nuclear periphery allowing Xist to spread into active genes. **(B)** Factors involved in Xist-mediated maintenance of gene silencing. PRC1 (mediating H2A119ub) and PRC2 (mediating H3K27me3) complexes are recruited by Xist. **(C)** Proteins mediating Xist spreading: SAf-A, CIZ1 and YY1. **(D)** Proteins implicated in RNA methylation and early gene-silencing.

Rbm15, a RRM-containing protein involved in RNA methylation, has also been shown to associate with the A-repeat of Xist (Cirillo et al., 2016). Comparative analysis of Rbm15 and SPEN eCLIP data (Cirillo et al., 2016), shows a very similar binding pattern, implying a competitive relationship. Partial overlapping function of SPEN and Rbm15 would explain the mild effects in gene de-repression observed in individual KO and KD *in vivo* models of the two factors (McHugh et al., 2015; Moindrot et al., 2015; Patil et al., 2016). A useful experiment that has yet to be performed would be a competition assay between SPEN and Rbm15 with Xist (Figures 2C, 3A).

More controversial is the interaction between certain subunits of the Polycomb2 (PRC2) complex (i.e., Ezh2 and Suz12) and Xist RNA (Figure 3B). In fact, literature reports evidence arguing in favor (Zhao et al., 2008; Kanhere et al., 2010) and against (Cerase et al., 2014; Almeida et al., 2017; Pintacuda et al., 2017) the idea that such interactions occur, or are critical, for PRC2 recruitment on the Xi. The main point of discussion is the observation of a strong interaction between the A-repeat and PRC2 *in vitro* (Delli Ponti et al., 2017; Lu et al., 2017), which seems to be dispensable *in vivo* (Almeida et al., 2017). However, while the Xist-PRC2 interaction *in vitro* is strong (Zhao et al., 2008; Kanhere et al., 2010), it is possible that *in vivo*, the interaction of the PRC2 complex to the A-repeat has very fast kinetics that prevents it to be captured in most studies relying on crosslinking (Sunwoo et al., 2015). On the other hand, *de novo* recruitment of PRC1 and PRC2 was observed to be mediated by a form of Xist lacking the A-repeat element in ESCs (da Rocha et al., 2014; Almeida et al., 2017).

This apparent contradiction could be reconciled envisioning a different pathway for PRC2 recruitment in undifferentiated compared to fully differentiated cells. In fact, recent evidence confirmed that in differentiating ES cells, Xist mediates PRC2 recruitment via PRC1-mediated H2A119 ubiquitination (Almeida et al., 2017), which therefore represents the fundamental *de novo* recruitment mechanism for Polycomb on the Xi. Notably, the fact that H3K27me3 accumulation on the Xi is entirely Xist dependent in fully differentiated cells (Kohlmaier et al., 2004), suggests that the Xist and perhaps the A-repeat may play a role in the maintenance of Polycomb rather than in its early establishment, reinforcing the *de novo* recruitment pathway. In the future, it will be crucial to systematically address these points, by quantitatively measuring the loss or redistribution of H3K27me3 marks in the absence of the A-repeat element.

#### B-repeat

The highly repetitive nature of GC-rich modules within the B-repeat region of Xist severely affects its mappability. In fact, highly repetitive sequences found within the genome are difficult to align and input into *in silico* studies (Kawaguchi and Kiryu, 2016). This consideration must be taken into account when critically assessing the lack of secondary structures reported through genome-sequencing based techniques. Nevertheless, the conservation of the B-repeats among mammals implies functionality, as recently confirmed in studies showing their role in binding to the nuclear matrix protein hnRNPK (Chu et al., 2015; Cirillo et al., 2016), and in recruiting the Polycomb repressive complex 1 (PRC1) to Xist-bound chromatin (da Rocha et al., 2014; Almeida et al., 2017; Pintacuda et al., 2017) (Figure 3B).

#### C-repeat

Xist C-repeat has an important role for Xist spreading and chromatin/matrix localization. Sarma et al. have highlighted the functional relevance of this repetitive region, using LNAs complementary to Xist C-repeat region, and showing a defect in localization of Xist, which was attributed to loss of binding of hnRNPU/Saf-A and YY1(Sarma et al., 2010). Indeed, targeting the LNA-4978 to the Xist C-repeats is predicted to completely disrupt its structure and displace Xist from chromatin (Figure 3C).

Most high-throughput studies, including that of Lu et al. (2016), failed to identify a specific folding in this region. However, as in the case of the B-repeat, this could be due to difficult mappability of this C-rich region. Scarce conservation between human and mouse both in terms of sequence and extension of this region, may suggest that perhaps the C-repeat element could have accumulated divergent functions during evolution.

#### E-repeat

**Figure 4:**
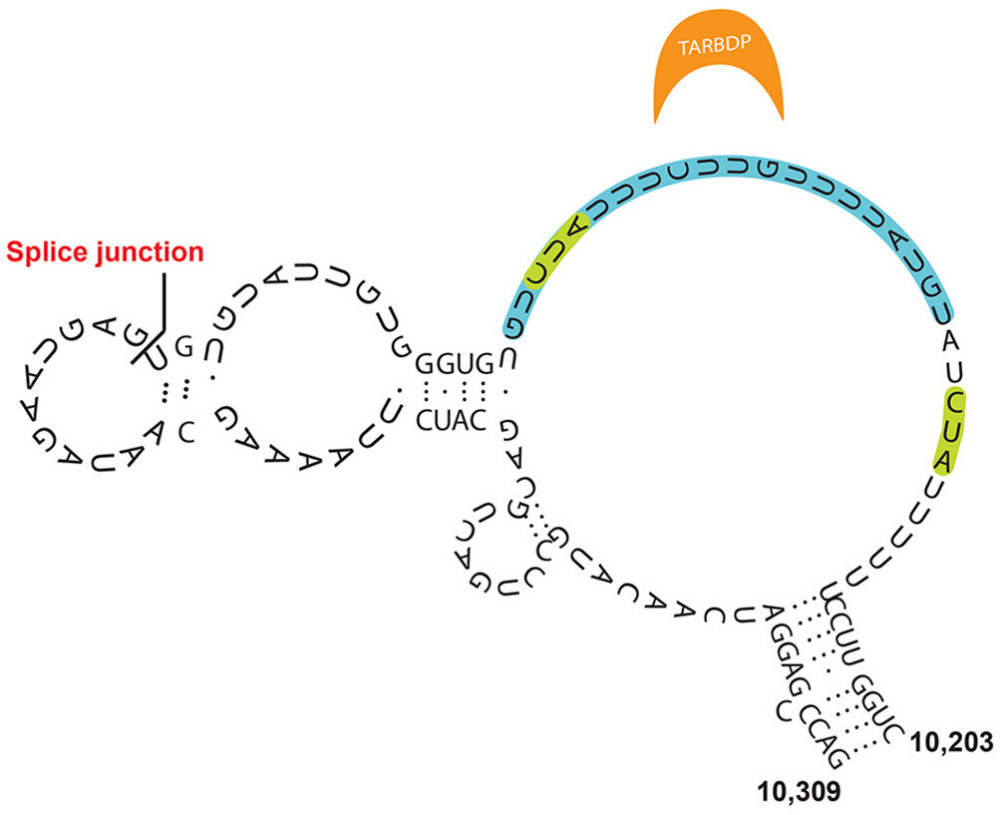
Model for Xist E-repeat structure (Smola et al., 2016). Binding sites for TARDBP, which are supported by SHAPE data, are indicated in green, while the CLIP supported binding site is indicated in blue.

Smola et al. observed that the RNA-binding proteins TARDBP, CELF1, PTBP1, previously implicated in a number of functional nuclear pathways, can bind to the E-repeat, although these factors are not necessary or redundant for XCI (McHugh et al., 2015). These proteins can only be stably retained when bound to the properly folded E-repeat element (Figure 4) (Smola et al., 2016). They suggest that PTBP1 and TARDBP interaction with the E-repeat is highly specific. Interestingly, the RNA binding proteins CELF1, and PTBP1 do not seem to bind Xist in a sequence-specific manner, but mostly along loosely structured regions. Another independent study also found PTBP1 as binding the E-repeat of Xist (Cirillo et al., 2016).

Ridings-Figueroa and colleagues, studied the interaction between the nuclear protein CIZ1 and Xist E-repeat (Ridings-Figueroa et al., 2017). In embryos, Xist localization was disrupted when CIZ1 was depleted. A similar result, was obtained by Sunwoo et al. (2017). This could be explained through the suggested interaction between CIZ1 and PTBP1(Ridings-Figueroa et al., 2017).

Very recently, the E-repeat was also associated with recruitment of the MLL/Set factor, ASH2L (Yue et al., 2017). Interestingly, deletion of the E-repeat generated in this study element increased expression of XCI escapees. More work needs to be done, in order to put this result in a functional context.

LBR has been reported to bind Xist RNA in proximity to the A- and E-repeats (Cirillo et al., 2016). Such binding plays a crucial role in mediating the localization of Xist to active genes and the tethering of the Xi territory to the nuclear periphery. According to the authors of the study, Xist localization and Xi positioning, result in the proper establishment of gene-silencing and its stabilization, respectively (Chen et al., 2016). However, it is likely that more factors, including SAF-A, are needed for Xist localization to the nuclear periphery or the nucleolus (Figure 3A).

#### Other Xist binding factors

SAF-A was one of the first characterized factors reported to accumulate on the Xi and directly interact with Xist RNA (Helbig and Fackelmayer, 2003; Hasegawa et al., 2010). Under particular experimental conditions, SAF-A was shown to be both sufficient and necessary for Xist spreading over the Xi and, consequently, for Xist-mediated gene silencing (Cerase et al., 2015; McHugh et al., 2015) (Figure 3C). It is possible—however—that more factors are needed for Xist spreading along the Xi territory. Indeed, Xist dependency on SAF-A may be limited to certain developmental stages and tissues (Kolpa et al., 2016). Furthermore, CLIP profiles, have revealed a broad interaction between SAF-A and Xist, potentially pointing toward a scenario of cell-to-cell variability in the binding pattern (Cirillo et al., 2016).

#### Xist m6A methylation, structure and gene-silencing

The combinatorial complexity of amino acid chains is matched by the number of possible modifications of the nucleotides, which have been described as existing in at least 100 post-transcriptionally modified states (van Delft et al., 2017). The post-transcriptional modifications can potentially be reversed during the life-span of the RNA, giving the primary sequence a dynamic nature. Recently, the N6 methylation of adenosine (m6A) was widely characterized in the transcriptome, and its functional role emerged in many cellular contexts (Dominissini et al., 2012; Meyer et al., 2012). m6A has been shown to be associated with mRNA stability; it also dictates lncRNA-protein accessibility and specificity (Dominissini et al., 2012; Ke et al., 2017; van Delft et al., 2017). Xist RNA was shown to be methylated in a handful of well-defined positions (Dominissini et al., 2012; Patil et al., 2016). It is likely that methylation of Xist RNA subtly alters its structure (Liu N. et al., 2017). However, it is not currently known how Xist m6A modifies its structure, nor how this may modulate protein-binding affinity (Zhou et al., 2016). It was recently proposed that Xist m6A modification could mediate recruitment of YTHDC1, a known reader of methylated RNA, and consequent transcriptional silencing through a yet-undefined mechanism (Figure 3D) (Patil et al., 2016). In the future, it will be crucial to clarify the contribution of m6A, as well as of other RNA modifications, to Xist structure(s).

## Final remarks

Xist RNA is the best-characterized lncRNA to date, and historically has been considered the paradigmatic example of a non-coding RNA regulating gene expression. So far, a number of different structures have been proposed for the Xist RNA molecule, each of which depends to a certain extent on the experimental conditions of the analysis (Duszczyk et al., 2008, 2011; Maenner et al., 2010; Duszczyk and Sattler, 2012; Fang et al., 2015; Lu et al., 2016; Smola et al., 2016; Liu F. et al., 2017). In fact, technical aspects intrinsic to each employed technique may have introduced biases in the final invoked model(s) (Shen et al., 1995; Latham et al., 2005; Low and Weeks, 2010; Somarowthu, 2016; Barra and Leucci, 2017).

For instance, the PARIS technique, provides *in vivo* data, and thus direct information about the dsRNA components of functional Xist molecules. However, this technique cannot detect whether the detected pairing arises from inter- or intra-Xist molecule base pairs.

On the opposite side, NMR studies produce high-resolution data of RNA tertiary structures, but cannot capture *in vivo* structures that are most likely mediated by protein interactions; additionally, they can only provide structural information for small, isolated and highly purified regions of the RNA at any one time. SHAPE, DMS, and SHAPE-MaP must rely on the aid of computational structure prediction and modeling to account for RNA secondary structures. In addition to the intrinsic biases of different techniques, some variability between proposed Xist structures also depends on the modeling constraints on raw data. In the future it will be essential to set common standards for structural modeling, also taking into account the dynamic nature of lncRNAs, which ultimately should be represented as ensembles of discrete interconverting conformations rather than rigid “averaged” structures.

Although structural studies on lncRNAs are still in their early days, combining models derived from different lines of research, can help to infer a general structure of Xist and identify well-supported features, especially when structural datasets were also tested by functional analyses.

First, results discussed in this review seem to suggest that the A-repeat element of Xist adopts an inter-repeat structure *in vivo*, which is essential for mediating gene silencing (Duszczyk et al., 2008, 2011; Lu et al., 2016). Similarly, other segments of Xist are likely to use inter-repeat binding to fold in 3D-space and mediate multimeric interactions with RNA-binding proteins. However, current data do not exclude the hypothesis that the modularity and 3D-conformation of Xist repeats is mediated or facilitated by unstructured or loosely structured intervening regions (Duszczyk et al., 2011; Minks et al., 2013; Chu et al., 2015).

Recent studies provided reproducible datasets of RNA-binding factors specifically interacting with Xist, and gave some insights into their function. However, for the most part, the molecular mechanism of their interplay with Xist remains unknown. A better understanding of Xist structure will be crucial to dissect the assembly of functional RNA-interactomes at the molecular level, and provide a paradigm for lncRNAs function, beyond the field of X chromosome inactivation.

Many other examples of fully functional, regulatory lncRNAs have been reported in the literature. Their biological function is exerted mostly through interaction with binding partners (mRNAs, ncRNAs and proteins), and is dependent on their structure (Mellin and Cossart, 2015; Aktaş et al., 2017). The ability of folding into stable structures confers regulatory RNAs three main advantages: (1) Decreased evolutionary constraints on the mutation rate of their primary sequences, (2) The generation of modular units that can be assembled independently to potentially recruit a combination of diverse molecular machineries; (3) Modulation of the strength of specific interactions by repeating one module or variations of that module (i.e., variable multimerization platform).

In this context, the convergent evolution case of Xist/Rsx becomes relevant. Rsx RNA is the metatherian analog of Xist (Grant et al., 2012). Both lncRNAs evolved independently to play a crucial role in X-inactivation of eutherian and marsupial mammals respectively, by recruiting the analogous gene-silencing machinery. Even if Xist and Rsx do not share any sequence analogy, they both have tandem repetitions that are possibly folded into similar structures, which may be involved in contacting orthologous chromatin remodeling machinery (Grant et al., 2012), arguing in favor of the idea that evolution tends to select for functional RNA folds over primary sequences.

## Author contributions

AC: conceived this project. AC, GP and AY designed the figures and wrote the paper.

### Conflict of interest statement

The authors declare that the research was conducted in the absence of any commercial or financial relationships that could be construed as a potential conflict of interest. The reviewer EGS and handling Editor declared their shared affiliation.

## Abbreviations

AMT: psoralen-derivative 4′-aminomethyltriosalen
SHAPE: 2′-hydroxyl acylation analyzed by primer extension
DMS: Dimethyl Sulfate
SHAPE-MaP: 2′-hydroxyl acylation analyzed by primer extension and mutagenesis profiling
PARIS: psoralen analysis of RNA interactions and structures
CLIP: Cross-linking Immuno precipitation
RIP: RNA-Immuno-Precipitation
PARS: Parallel Analysis of RNA Structure
CROSS: Computational Recognition of Secondary Structure
FRET: Förster Resonance Energy Transfer.
